# Mechanisms of phototherapy of Alzheimer’s disease during sleep and wakefulness: the role of the meningeal lymphatics

**DOI:** 10.1007/s12200-023-00080-5

**Published:** 2023-09-18

**Authors:** Semyachkina-Glushkovskaya Oxana, Shirokov Alexander, Blokhina Inna, Fedosov Ivan, Terskov Andrey, Dubrovsky Alexander, Tsoy Maria, Elovenko Daria, Adushkina Viktoria, Evsukova Arina, Telnova Valeria, Tzven Anna, Krupnova Valeria, Manzhaeva Maria, Dmitrenko Alexander, Penzel Thomas, Kurths Jürgen

**Affiliations:** 1 Institute of Physics, Humboldt University, Berlin, 12489 Germany; 2 Department of Biology, Saratov State University, Saratov, 410012 Russia; 3 Institute of Biochemistry and Physiology of Plants and Microorganisms, Russian Academy of Sciences, Saratov, 410049 Russia; 4 Charité – Universitätsmedizin Berlin, Berlin, 10117 Germany; 5 Department of Complexity Scienc, Potsdam Institute for Climate Impact Research, Potsdam, 14473 Germany

**Keywords:** Alzheimer’s disease, Photobiomodulation, Brain lymphatics, Sleep, Mechanisms

## Abstract

With the increase in the aging population, the global number of people with Alzheimer’s disease (AD) progressively increased worldwide. The situation is aggravated by the fact that there is no the effective pharmacological therapy of AD. Photobiomodulation (PBM) is non-pharmacological approach that has shown very promising results in the therapy of AD in pilot clinical and animal studies. However, the mechanisms of therapeutic effects of PBM for AD are poorly understood. In this study on mice, we demonstrate that photodynamic effects of 5-aminolevulenic acid and laser 635 nm cause reduction of network of the meningeal lymphatic vessels (MLVs) leading to suppression of lymphatic removal of beta-amyloid (Aβ) from the right lateral ventricle and the hippocampus. Using the original protocol of PBM under electroencephalographic monitoring of wakefulness and sleep stages in non-anesthetized mice, we discover that the 7-day course of PBM during deep sleep vs. wakefulness provides better restoration of clearance of Aβ from the ventricular system of the brain and the hippocampus. Our results shed light on the mechanism of PBM and show the stimulating effects of PBM on the brain lymphatic drainage that promotes transport of Aβ via the lymphatic pathway. The effects of PBM on the brain lymphatics in sleeping brain open a new niche in the study of restorative functions of sleep as well as it is an important informative platform for the development of innovative smart sleep technologies for the therapy of AD.

**Graphical Abstract:**

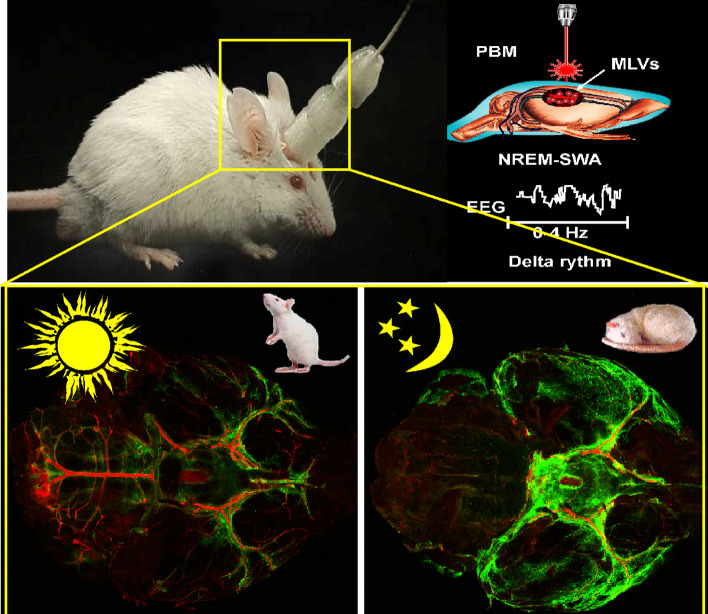

## Introduction

Alzheimer’s disease (AD) is a progressive brain disease beginning with memory loss and leading to problems with thinking, language, disorientation, and behavior. From 1990 to 2019, the incidence of AD increased by 147% [1–3]. It has been reported that the global number of aged people with AD doubles every five years [4]. Based on the data obtained from the 2019 Global Burden of Disease database, the incidence of AD will triple by 2060 [4]. However, the pharmacological therapies of AD have failed to show effectiveness and safety [5–8]. Indeed, ENGAGE phase 3 randomized clinical trials of Aducanumab obtained on 3285 patients with AD report different consequences associated with therapy, including edema (35.2%), headache (46.6%), confusion (14.6%), dizziness (10.7%), nausea (7.8%) and microhemorrhage (19.1%) and superficial siderosis (14.7%) [8]. Therefore, the search for non-pharmacological treatments for AD is necessary.

Photobiomodulation (PBM) is non-pharmacological approach based on the use of red or near-infrared light that has shown very promising results in the therapy of AD in pilot clinical and animal studies [9–21]. The Food and Drug Administration (FDA) recognized PBM as safe because PBM is a non-invasive method without any side-effects. There is evidence that the increase in metabolism and microcirculation of brain tissues as well as reduction of oxidative stress and inflammation are the mechanisms of therapeutic effects of PBM [12, 17, 18, 20]. It was recently discovered that PBM effectively stimulates lymphatic removal of wastes and toxins, including amyloid-β (Aβ), from the brain [21–29].

The important role of the meningeal lymphatic vessels (MLVs) in the clearance of Aβ from the central nervous system (CNS) we discussed in our recent review [21]. The Italian anatomist Mascagni discovered the lymphatic network of transparent vessels in the brain meninges of humans in the eighteenth century [30]. He called them MLVs. However, for two centuries no one was able to repeat his discoveries [31–33]. Therefore, in science it was believed that there were no MLVs. In 1988, Aβ depositions were first identified in the meninges of patients with AD [34]. Since science was dominated by dogma from the absence of MLVs, the cerebrovascular basement membrane was seen as a key pathway of Aβ clearance from the CNS [35]. Only in 2015, when MLVs were re-discovered in the meninges of rodents and humans along the main cerebral veins and the middle meningeal artery, a growing number of results clearly showed that MLVs are tunnels for clearance of Aβ from the brain [22, 28, 36–38].

The brain lymphatic drainage is increased during deep sleep [21, 22, 39–42]. Xie et al. for the first time reported that clearance of Aβ is observed only during non-rapid eye movement (NREM) sleep, while wakefulness is accompanied suppression of removal of this toxin from the mouse brain [39]. Later, we demonstrated that PBM during deep sleep vs. wakefulness provides better therapy of AD in mice [22]. Currently, sleep is considered as an informative platform for the development of promising therapeutic approaches for the AD pathology [21, 41–44]. However, the question of why PBM during sleep is more effective in therapy of AD than during wakefulness remains poorly understood. In this research, we studied the role of MLVs in the PBM-related stimulation of removal of Aβ from the mouse brain during deep sleep and wakefulness.

## Methods

### Subjects

Male C57BL/6 mice (25–28 g, 3 months age) were used in all experiments and were obtained from the National Laboratory Animal Resource Centre in Pushchino (Moscow area, Russia). The animals were housed under standard laboratory conditions with access to food and water ad libitum. All experimental procedures were performed in accordance with the “Guide for the Care and Use of Laboratory Animals”, Directive 2010/63/EU on the Protection of Animals Used for Scientific Purposes, and the guidelines from the Ministry of Science and High Education of the Russian Federation (Nº 742 from 13.11.1984), which have been approved by the Bioethics Commission of the Saratov State University (Protocol No. 8, 18.04.2023). The mice were housed at (25 ± 2)°C, 55% humidity, and 12:12 h light–dark cycle (light: 08:00 am–08:00 pm). The mice adapted to the experimental conditions during one week before the beginning of the experiments to ensure acclimation to the housing room of the animal facility. The experiments were performed in the following groups: (1) Laser; (2) 5-Aminolevulinic acid (5-ALA); (3) 5-ALA + Laser; (4) PBM during sleep; (5) PBM during wakefulness, *n* = 4–6 in each group in all sessions of the experiments.

**Fig. 1:**
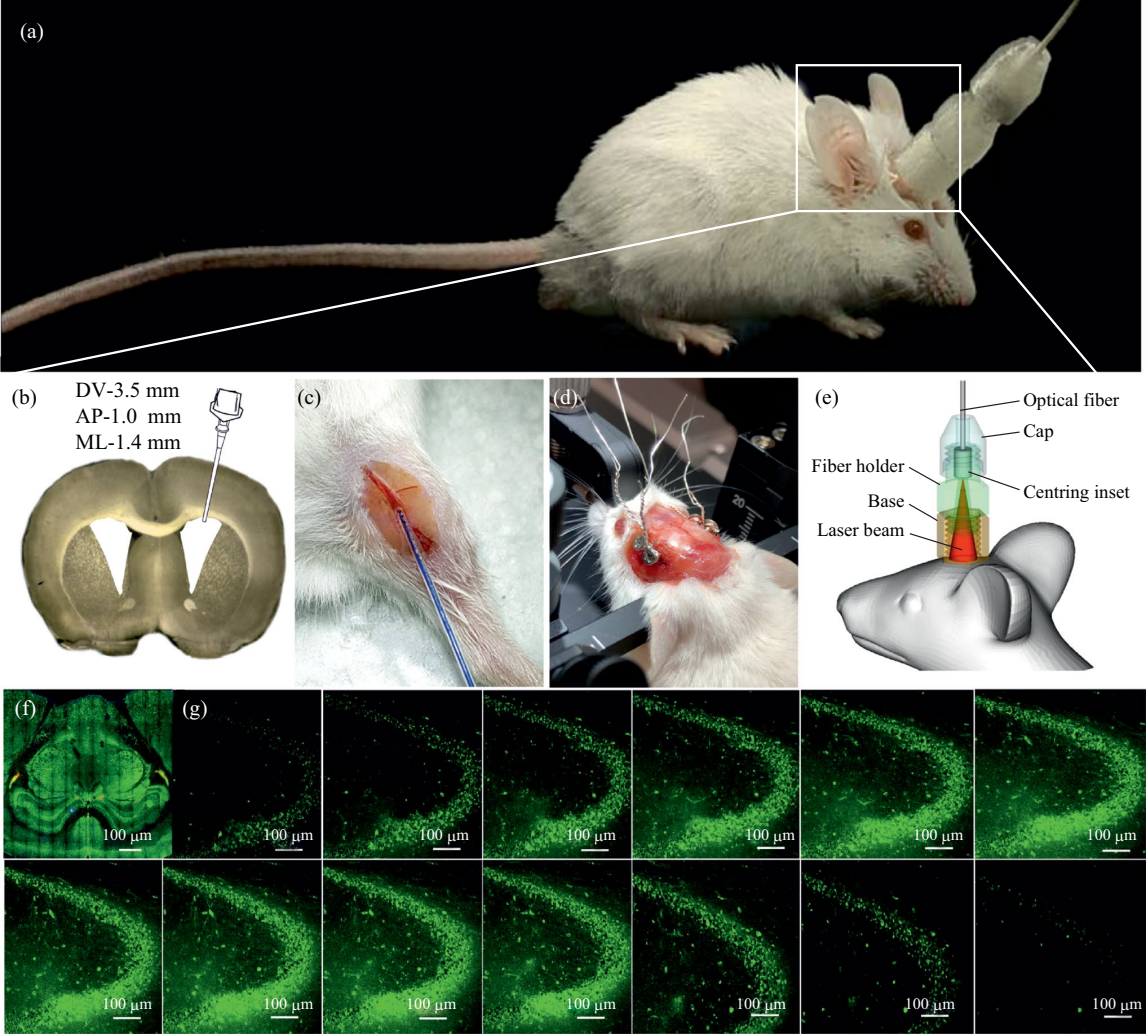
Preparations of mice for the experiments and the analysis of presence of FAβ in the hippocampus: **a** View of the mouse before the experiments; **b** implantation of chronic polyethylene catheters into the right lateral ventricle for the injection of fluorescent Aβ and **c** in the femoral vein for the Evans Blue dye injection; **d** implantation of the EEG electrodes into the cortex; **e** fixation of head plate with LED; **f** representative image of brain slice with selected region of interest (SROI, place of Aβ injection) in the hippocampus; **g** representative images of spreading of FAβ in the hippocampus through the entire depth (50 µm) of one slice.

Figure 1 illustrates preliminary preparations of mice: ten days before experiments, surgical procedures were performed, including (a) implantation of chronic polyethylene catheters into the right lateral ventricle for the injection of fluorescent Aβ (FAβ) and (b) in the femoral vein for the Evans Blue dye injection; (c) implantation of the EEG electrodes into the cortex; (d) preparation of the chronic cranial optical window.

### Electroencephalography (EEG)

A two-channel cortical EEG/one-channel electromyogram (Pinnacle Technology, Taipei, China) was recorded. The mice were implanted with two silver electrodes (tip diameter: 2–3 µm) located at a depth of 150 µm in coordinates (*L*: 2.0 mm and *P*: 2 mm) from the bregma on either side of the midline under inhalation anesthesia with 1% isoflurane (Sigma-Aldrich, St Luis, USA, at rate 1 L/min N_2_O/O_2_—70/30 ratio). The head plate was mounted and small burr holes were drilled. Afterward, EEG wire leads were inserted into the burr holes on one side of the midline between the skull and the underlying dura. EEG leads were secured with dental acrylic (Zhermack SpA, Badia Polesine, Italy). An EMG lead was inserted in the neck muscle. Ibuprofen (Bhavishya Pharmaceuticals Pvt. Ltd., Hyderabad, Telangana, India, 15 mg/kg) for the relief of postoperative pain was provided in their water supply for 2 to 3 days prior to surgery and for 3 days post-surgery. The mice were allowed 10 days to recover from surgery prior to beginning the experiment.

**Fig. 2:**
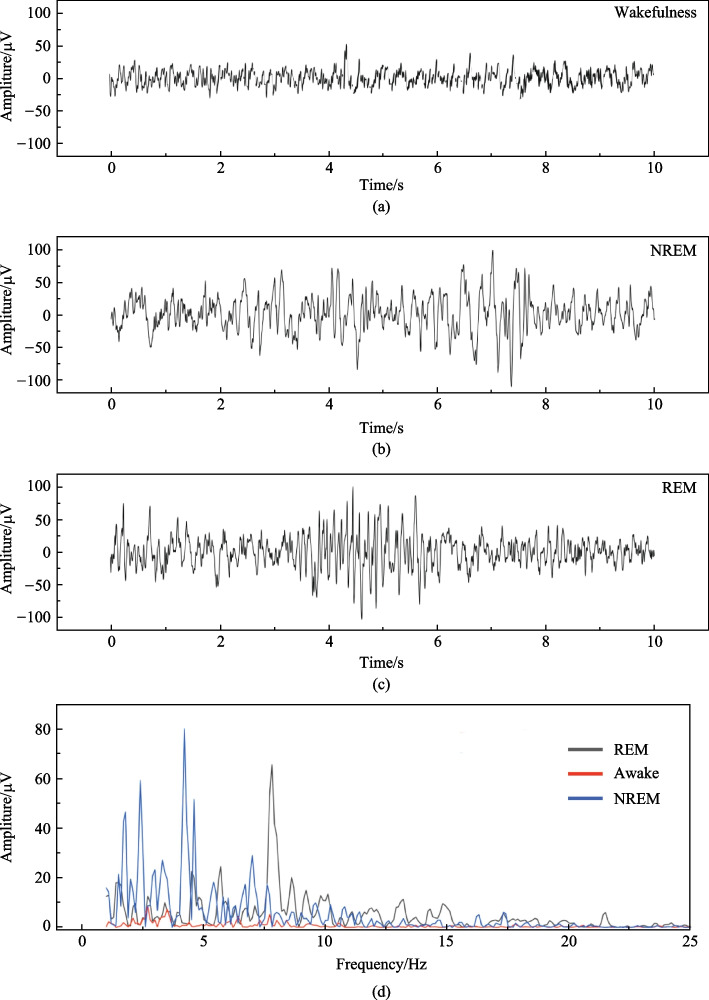
EEG analysis of the brain activity in mice: **a**–**c** EEG patterns of wakefulness, NREM and REM sleep, respectively; **d** EEG signal spectrum characteristics for wakefulness, NREM and REM sleep

Wakefulness, NREM and rapid eye movement (REM) sleep were defined as described in our previous study [22]. Briefly, wakefulness was defined as a desynchronized EEG with low-amplitude and high-frequency dynamics (> 10%, 8–12 Hz) and relatively high-amplitude EMG. NREM sleep was recognized as synchronized activity with high amplitude, which is dominated by low-frequency delta waves (0–4 Hz) comprising > 30% of EEG waveforms/epoch and a lower-amplitude EMG. REM was identified by the presence of theta waves (5–10 Hz) comprising > 20% of EEG waveforms/epoch with a low EMG amplitude. Figure 2 demonstrates the EEG patterns and spectrum characteristics of wakefulness, NREM and REM sleep in mice.

### Photo-ablation of MLVs

To ablate MLVs, we used photoexcitation of the 5-aminolevulinic acid (5-ALA) by laser 635 nm. The mice from the 5-ALA + Laser group were anesthetized with 1% isoflurane (Sigma-Aldrich, St Luis, USA, at rate 1 L/min N_2_O/O_2_—70/30 ratio) and 5-ALA (Niopik, Moscow, Russia, 5 μL) was injected into the cisterna magna at a speed of 0.1 μL/min. Fifteen minutes later, a laser (XPeBRD-L1-0000-00901, CREE, Inc.), which emits a 1 W maximum light power at 635 nm with a light dose of 15 J/cm^2^ was applied through the skull in different places, including the cisterna magna, the left and right transverse sinuses, the superior sagittal sinus, and the junction of all sinuses.

In the control groups, mice were injected with 5-ALA only (5 μL, into the cisterna magna) or were treated by laser 635 nm without 5-ALA. The effects of photo-damages of MLVs was evaluated as % LYVE-1-positive vessels (the marker of the lymphatic endothelium) coverage of the venous sinuses (Fig. 6). In all groups, 4 days after photo-ablation of MLVs, FAβ was injected into the hippocampus and 3 days after that the brains and the meninges were removed for confocal microscopy.

### Effects of PBM during sleep and wakefulness on restoration of brain lymphatic drainage and clearance of FAβ from the hippocampus

An amount of 1 μL of FAβ at a final concentration of 1 μg or 0.2 nM^1^^)^, HiLyte™ Fluor 488 labeled (AnaSpec Inc., Fremont, California, USA) was injected into the hippocampus (AP—2.0 mm; ML + /–1.3 mm; DV—1.9 mm) or 5 μL of FAβ at a final concentration of 5 μg or 1 nM was injected into the right lateral ventricle (AP—1.0 mm; ML—1.4 mm; DV—3.5 mm) at a rate of 0.1 μL/min using microinjector (Stoelting, St. Luis, USA) with a Hamilton syringe with a 29-G needle (Hamilton Bonaduz AG, Switzerland). The implantation of chronical catheter a polyethylene catheter (PE-10, 0.28 mm ID × 0.61 mm OD, Scientific Commodities Inc., Lake Havasu City, Arizona, USA) into the right lateral ventricle was preformed according to the protocol reported by DeVos and Miller [45].

The PBM was performed with 3835 SMD LED (central wavelength 1050 nm and spectrum width of 50 nm). LED was operated in continuous wave mode with output power of 50 mW that was distributed over 3.6 mm spot at skull surface. It was attached to a 3D printed head plate and connected with 0.5 m long flexible wires to driver module. The irradiance at skull surface does not exceed 0.5 W/cm^2^. Dose for single 17 min procedure each day was 500 J/cm^2^ and for 7 days treatment it was 3.5 kJ/cm^2^.

The head plate with LED was fixed in the region of the parietal and interparietal bones using dental acrylic (Zhermack SpA, Badia Polesine, Italy) under inhalation anesthesia with 1% isoflurane (Sigma-Aldrich, St Luis, USA, at rate 1 L/min N_2_O/O_2_—70/30 ratio). The LED was fixed to the head plate with two screws.

**Fig. 3:**
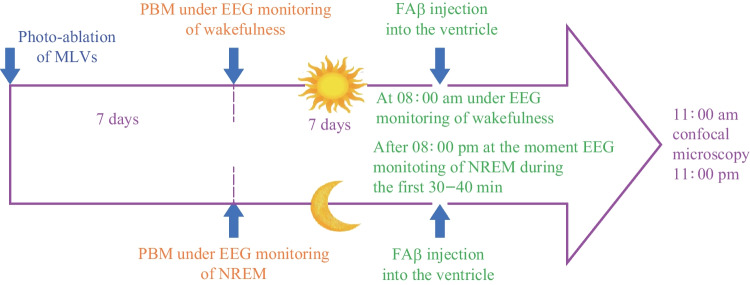
Design of the study of the PBM effects on distribution of FAβ in the brain fluid system during wakefulness and deep sleep after photo-ablation of MLVs

**Fig. 4:**
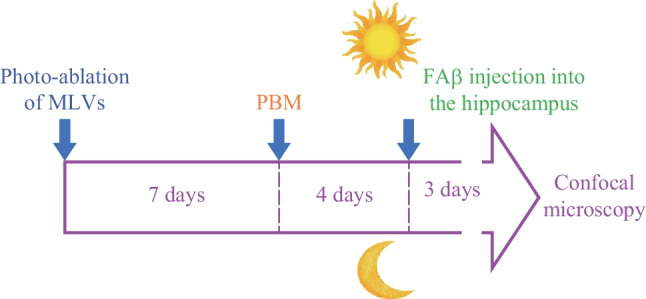
Design of the study of the PBM effects on lymphatic removal of FAβ from the hippocampus during wakefulness and deep sleep after photo-ablation of MLVs

The PBM performed daily during one week under EEG monitoring of wakefulness or NREM, 7 days after photo-ablation of MLVs (Figs. 3 and 4).

To study the PBM effects during wakefulness and deep sleep on the recovery of FAβ distribution in the brain fluid system, the intracerebroventricular injection of FAβ was performed at 08:00 am under the EEG monitoring of wakefulness and after 08:00 pm at the time of EEG monitoring of NREM during the first 30 min of observation (Fig. 4). The time of 8 am and 8 pm for the intracerebroventricular injection of FAβ was chosen due to the light regime of vivarium and to standardize the protocol to start the experiment at the time of the natural transition to sleep or awakening. To keep the same time for the distribution of FAβ in the waking and sleeping states, mice that did not show NREM during the first 30 min of observation were not included in the studies.

The ex vivo optical study of FAβ distribution in the brain fluid system was performed 3 h after the intracerebroventricular injection of FAβ (Fig. 4). Afterward, the whole brain imaging from dorsal aspect was performed using confocal microscopy.

**Fig. 5:**
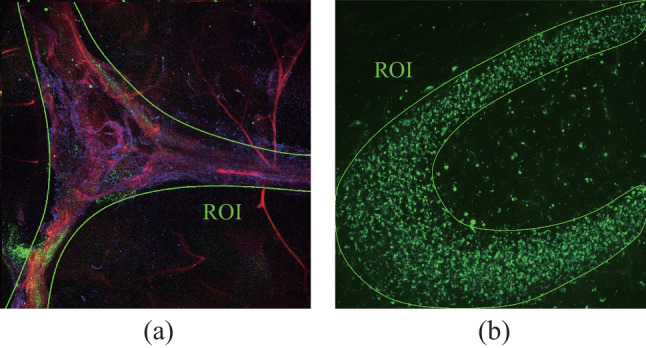
Illustration of SROIs for the statistical analysis of the FAβ presence in the meninges (**a**) and in the hippocampus (**b**)

To analyze the PBM effects during wakefulness and deep sleep on the recovery of FAβ removal from the hippocampus after photo-ablation of MLVs, FAβ was injected into it in the middle of PBM course, i.e., on 4th day of PBM (Fig. 5). Thus, ex vivo optical study of the presence of FAβ in the areas CA1c, CA2 and CA3a-c of hippocampus was performed in mice receiving PBM during one week under EEG monitoring of wakefulness or NREM (Fig. 5).

The imaging was performed using a homemade optical instrument based on the monochrome camera acA2040–2090 μm (Basler, Ahrensburg, Germany) and a 50 mm 2.8 C-mount CCTV objective lens (Tamron, Japan). The lens was attached to the camera with a 15 mm extension tube to ensure macro imaging with a 23.3 to 31.8 mm field of view depending of the lens focusing ring adjustment. The lens was mounted on the vertical manual translation stage (Standa, Lithuania) above a Petri dish, where samples were submerged in a buffer solution. The top surface of each sample was covered with a 25 mm × 50 mm × 0.17 mm cover glass. The slider with filter sets (49,019, 49,002, Chroma Technology, USA) was placed just below the objective lens. Each filter set was illuminated with homemade condensers with 1W LEDs (635 nm for 49,019 and 460 nm for 49,002) to ensure uniform illumination over the camera field of view. Led illuminators were synchronized with the camera “fire” output.

The camera resolution was 2048 × 2048 pixels at 12 bit grayscale. Images were acquired in a dark room at a constant exposure time of 200 ms, and other settings were kept unchanged for all samples. Image acquisition and processing were performed with a custom software developed using the NI Vision and LabVIEW software (National Instruments, Eagan, USA) and the Fiji open-source image processing package [46]. Image processing procedures were identical for each pair of images (control and laser-treated samples) for each channel to ensure an accurate comparison of the fluorescence intensity.

The PBM performed daily during 1 week, 7 days after photo-ablation of MLVs and 3 days after the FAβ injection into the hippocampus (Fig. 5). The PBM carried out in the waking state or during deep sleep in the time of EEG monitoring of NREM (Fig. 2a and b).

### Immunohistochemistry (IHC)

The Evans blue dye (1%, Sigma-Aldrich, St Luis, USA) was intravenously injected 30 min before decapitation. For confocal imaging of presence of FAβ in the meninges and the hippocampus, we used the protocol for the IHC analysis with the markers for the lymphatic vessel endothelial hyaluronan receptor 1 (LYVE1) and for the blood endothelium CD31. The brain and the meninges were collected and free-floating sections were prepared. The tested tissues were fixed for 48 h in a 4% saline solution-buffered formalin. Afterward, sections of tissues were cut on a vibrotome (Leica, Wetzlar, Germany) with a thickness of 40–50 μm. The antigen expression was evaluated on sections of the mouse brain and the meninges according to the standard method of simultaneous combined staining of the drug (abcam protocols for free-floating sections) using a confocal laser scanning microscope (Nikon A1R MP, Nikon Instruments Inc., Tokyo, Japan). The nonspecific activity was blocked by 2 h incubation at room temperature with 10% BSA in a solution of 0.2% Triton X-100 in PBS. Solubilization of cell membranes was carried out during 1 h incubation at room temperature in a solution of 1% Triton X-100 in PBS. Incubation with primary antibodies in a 1:500 dilution took place overnight at 4 °C: with rabbit anti- LYVE-1 antibody (1:500; ab 218535, Abcam, Cambridge, UK) and rat antibodies to CD31 (1:500; ab 7388; Abcam, Biomedical Campus Cambridge, Cambridge, UK). At all stages, the samples were washed 3–4 times with 5-min incubation in a washing solution. After that, the corresponding secondary antibodies goat anti-rabbit IgG (H + L) Alexa Flour 555 and goat anti-rat IgG (H + L) Alexa Flour 405 (Invitrogen, Molecular Samples, Eugene, Oregon, USA) were applied. The blue-fluorescent DAPI nucleic acid used for staining nucleus of cells. The excitation maximum for DAPI bound to dsDNA is 358 nm, and the emission maximum is 461 nm. At the final stage, the sections were transferred to the glass and 15 µL of mounting liquid (50% glycerin in PBS) was applied to the section. The preparation was covered with a cover glass and confocal microscopy was performed.

### Quantitative analysis of confocal images

To analyze Aß in the areas CA1c, CA2 and CA3a-c of hippocampus of the brain, single 50 microns thick slice was taken in distance 150 microns anteriorly from the edge of the wound caused by microinjector needle. The confocal images of SROI (place of Aβ injection) in the hippocampus included 3D slice containing 10–14 images through depth of slice per each mouse (Fig. 1f and g). Since the hippocampal size and volume are highly variable among mice, we selected only one ROI with most representative results.

**Fig. 6:**
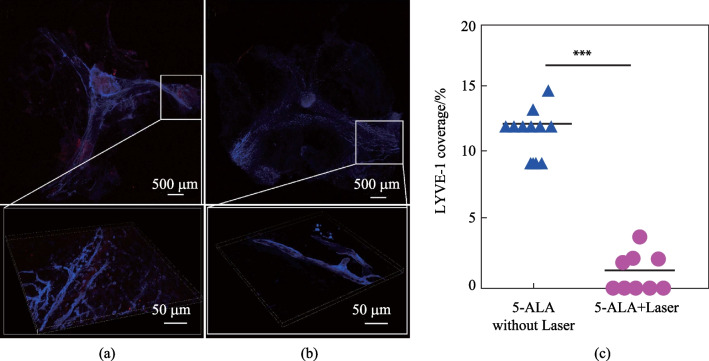
Network of MLVs around the venous sinuses: **a** before and **b** after photo-ablation of MLVs; **c** quantitative analysis of the LIVE-1 positive vessels coverage of the venous sinuses and expressed in %, *n* = 12 in the 5-ALA without Laser group and *n* = 9 in the group of 5-ALA + Laser, the Mann–Whitney U test

The SROI for calculation the integral characteristic of the intensity in the FAβ green channel was chosen as shown in the Fig. 6a. The boundary was drawn taking into account the morphology so that the entire sinus fell into SROI. At the same time, region of the cerebellum was excluded from consideration due to the high intragroup dispersion of the intensity of this zone. The areas at the edges of the brain (edges of the hemispheres) were not excluded due to the low intensity variability between all groups. The resulting intensity value was normalized to the SROI area. As a result, the integral characteristic of the intensity density was calculated, the contribution to which is mainly made by changes in the sinus.

In the case of analysis of area along the sagittal sinus, the area was selected using morphometric information about the location of MLVs (Fig. 5a) [36, 37]. In the case of analysis of the hippocampus, the area of accumulation of FAβ was well detected and the region of interest was chosen according to the area of maximum FAβ density and its homogeneity (Fig. 5b).

The FAβ analysis was included several steps:

The background subtraction by using a “rolling ball” algorithm [47].

The image binarization by threshold method [48];

The FAβ detection using shape and size information. The areas with a value of “1” were approximated by ellipses and an additional filter was applied to exclude areas that did not fall within the range from 15 to 300 μm^2^ area and in the Roundness values range [0.2; 1];

The analysis of FAβ in a segmented image: calculation of the proportion of the area occupied by particles (Sp) relative to the area of SROI: SS = (Sp/SROI)*100%;

The statistical analysis using the Wilcoxon rank sum test with continuity correction (Mann–Whitney–Wilcoxon test). The significance level was set at *p* < 0.05.

## Results

### Photodynamic ablation of MLVs

To ablate MLVs, we used 5-ALA and laser 635 nm. 5-ALA is widely used as a drug for photodynamic therapy (PDT) of brain tumors [49–52]. PDT uses a drug that is activated by light, called a photosensitizer, to kill cancer cells. By itself, 5-ALA is not a photosensitizer and does not accumulate in cells, however, when administered exogenously, it temporarily “overloads” the normal pathway of heme biosynthesis. Due to the reduced activity of the rate-limiting enzyme ferrochelatase, as well as iron deficiency in the tumor tissue, in contrast to the normal one, there is an accumulation of an intermediate product of biosynthesis–protoporphyrin IX (PP IX). Thus, 5-ALA is a precursor of the endogenous photosensitizer PP IX. PP IX is a rather active photosensitizer due to the presence of an intense absorption band with a maximum at a wavelength of 635 nm and the ability to efficiently generate singlet oxygen and reactive oxygen species providing photo-injury of vessels, including MLVs [53–55].

Figure 6 clearly demonstrates the effective photo-injury of MLVs by 5-ALA-PDT in all mice. Indeed, the coverage of LYVE-1 positive vessels along the sagittal and transverse sinuses was 5.7-fold lesser (*p* < 0.001) in the 5-ALA + Laser (photo-ablation, *n* = 9) vs. the 5-ALA without Laser (control, *n* = 12) groups. Thus, 5-ALA-PDT caused significant damages of MLVs in 100% of mice leading to significant reducing their network in the meninges of mice.

### Effects of 7 days-course of PBM during sleep and wakefulness on restoration of brain lymphatic drainage after the MLV damage

**Fig. 7:**
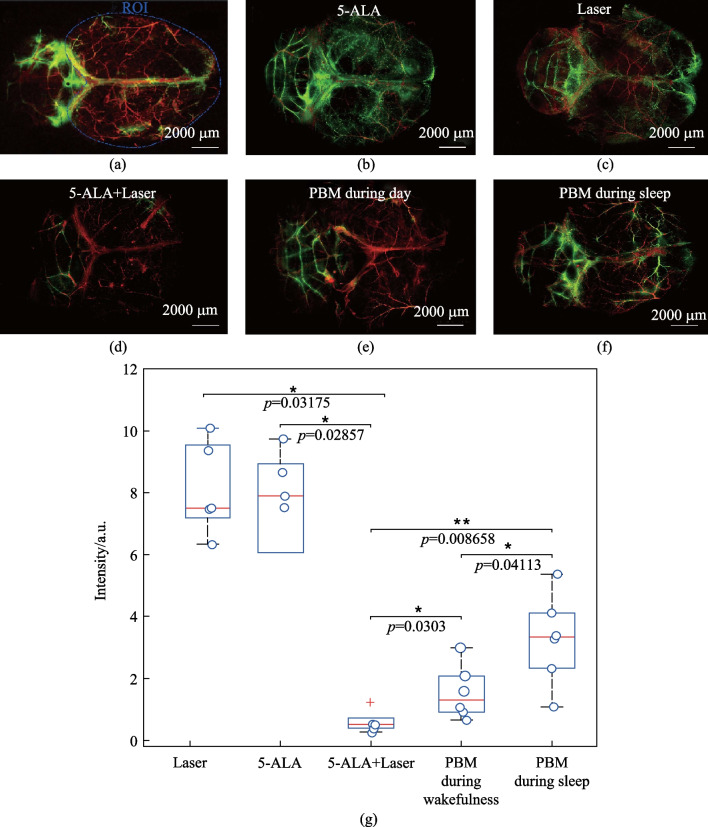
PBM effects during sleep and wakefulness on recovery of FAβ distribution in the brain fluid system after photo-ablation of MLVs: **a** SROI for the quantitative analysis of FAβ distribution in the brain fluid system; **b–f** confocal images of whole brain from its dorsal part demonstrating FAβ (green) spreading in the brain fluids in the tested groups. The blood vessels (red) were filled with Evans Blue dye; **g** quantitative analysis of FAβ distribution in the brain fluid system in the tested groups (*n* = 5 in the groups “Laser,” “5-ALA,” “5-ALA + Laser”; *n* = 6 in the groups “PBM during wakefulness” and “PBM during sleep”), the Mann–Whitney U test

In the first step, we answered the question whether PBM could help restore brain lymphatic drainage after the MLV damage. Figures 7b–d and g clearly demonstrate that photo-ablation of MLVs was accompanied by significant suppression of FAβ distribution in the brain fluid system compared with the controls. Indeed, the intensity of fluorescent signal from FAβ was 17.11-fold lesser in the 5-ALA + Laser group (photo-injury of LMVs) vs. the Laser group (0.44 ± 0.06 a.u. vs. 7.53 ± 0.66 a.u., *p* = 0.03175, *n* = 5 in each group, the Mann–Whitney U test) and was 19.22-fold lesser in the 5-ALA + Laser group vs. the 5-ALA group (0.44 ± 0.06 a.u. vs. 8.46 ± 0.49 a.u., *p* = 0.02857, *n* = 5 in each group, the Mann–Whitney U test). Figure 5a illustrates the region of SROI used for the statistical analysis of the intensity signal from FAβ in the brain fluid system. The choice of SROI is explained by the fact that MLVs located along the transverse and sagittal sinuses play a key role in brain drainage as well as because photo-ablation destroys MLVs in this area of the meninges [37]. Altogether, these results confirm that the MLV injury is associated with a significant decrease of brain lymphatic drainage.

Figures 7e–g show that the 7 days-course of PBM improved FAβ spreading in the brain fluids that was more pronounced in the group “PBM during sleep” vs. the group “PBM during wakefulness”. So, after PBM the intensity of fluorescent signal from FAβ was 7.43-fold higher in the group “PBM during sleep” vs. the group “5-ALA + Laser” (3.27 ± 0.64 a.u. vs. 0.44 ± 0.06 a.u., *p* = 0.008658, *n* = 6 in the group “PBM during sleep” and *n* = 5 in the group “5-ALA + Laser”) and was 3.52-fold greater in the group “PBM during wakefulness” vs. the group “5-ALA + Laser” (1.55 ± 0.38 a.u. vs. 0.44 ± 0.06 a.u., *p* = 0.0303, *n* = 6 in group “PBM during wakefulness,” *n* = 5 in the group “5-ALA + Laser,” the Mann–Whitney U test). Thus, brain lymphatic drainage was 2.11-fold higher in mice after the course of PBM during sleep vs. wakefulness (3.27 ± 0.64 a.u. vs. 1.55 ± 0.38 a.u., *p* = 0.04113, *n* = 6 in each group, the Mann–Whitney U test).

### Effects of 7 days-course of PBM during sleep and wakefulness on restoration of clearance of FAβ from the brain after the MLV damage

**Fig. 8:**
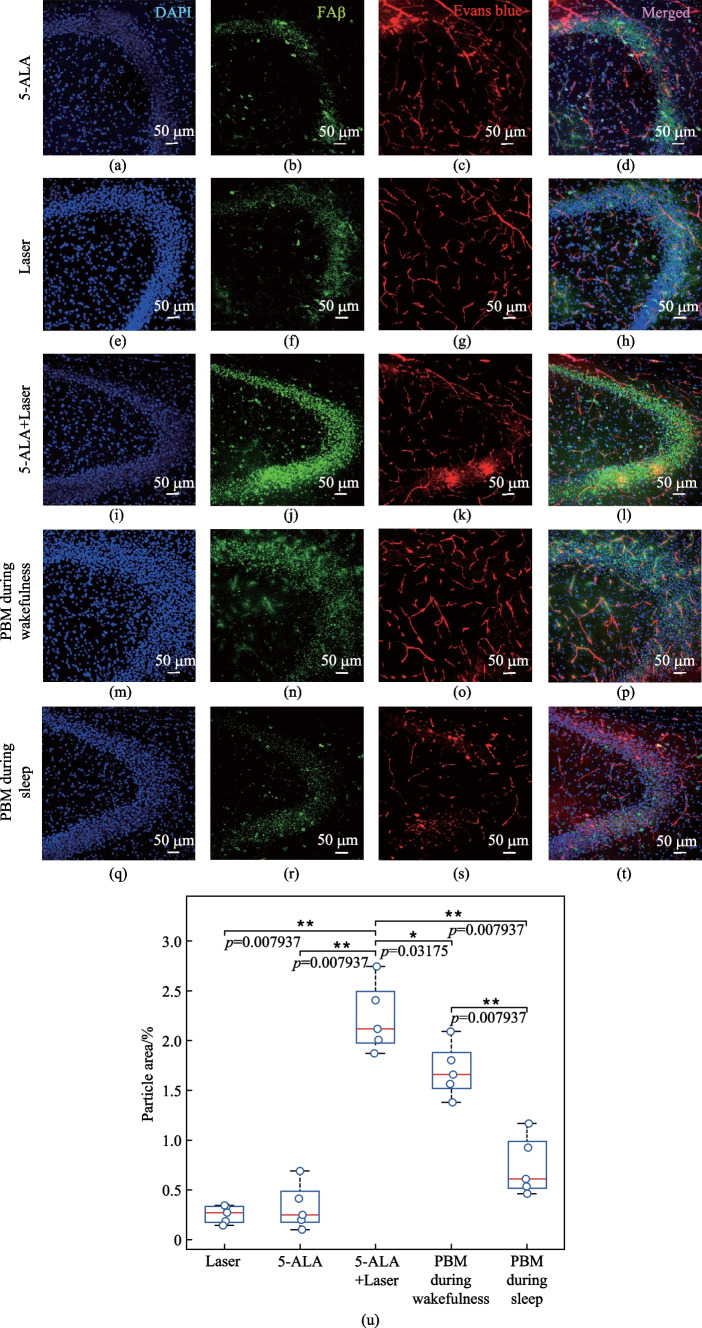
Qualitative and quantitative analysis of the PBM effects during sleep and wakefulness on recovery of evacuation of Aβ from the areas CA1c, CA2 and CA3a-c of hippocampus after photo-ablation of MLVs: **a**–**d** in the 5-ALA group; **e**–**h** in the Laser group; **i**–**l** in the 5-ALA + Laser group; **m**–**p** in the PBM during sleep group; **q**–**t** in the PBM during wakefulness group; **u** quantitative analysis of fluorescent signal from FAβ in the hippocampus in the tested groups, *n* = 5 in each group, the Mann–Whitney U test

In the next step, we studied the 7 days-course of PBM during sleep and wakefulness on the FAβ evacuation from the hippocampus after the MLV injury. Figures 8a–l and u demonstrate that photo-ablation of MLVs suppressed the FAβ evacuation from the hippocampus. Therefore, the FAβ hippocampal level was 6.72-fold and 8.88-fold higher in mice with the damaged MLVs compared with the controls, including the groups “5-ALA” and “Laser,” respectively (2.22 ± 0.15 a.u. vs. 0.33 ± 0.10 a.u., *p* = 0.007937 between the groups “5-ALA” and “5-ALA + Laser”; 2.22 ± 0.15 a.u. vs. 0.25 ± 0.03 a.u., p = 0.007937, between the groups “Laser” and “5-ALA + Laser,” *n* = 5 in each group, the Mann–Whitney U test).

Both PBM courses during sleep and in the waking state provided restoration of FAβ elimination from the hippocampus (Fig. 8m–u). Indeed, the intensity fluorescent signal from FAβ in the hippocampus was 3.04-fold and 1.31-fold lesser in the groups “PBM during sleep” and “PBM during wakefulness” vs. the group “5-ALA + Laser,” respectively (0.73 ± 0.13 a.u. vs. 2.22 ± 0.15 a.u., *p* = 0.007937 between the groups “PBM during sleep” and “5-ALA_Laser”; 1.69 ± 0.11 a.u. vs. 2.22 ± 0.15 a.u., *p* = 0.007937 between the groups “PBM during wakefulness” and “5-ALA_Laser”; *n* = 5 in each group, the Mann–Whitney U test). However, the FAβ hippocampal level after the course of PBM during sleep was 2.31-fold lesser than after the PBM course during wakefulness (0.73 ± 0.13 a.u. vs. 1.69 ± 0.11 a.u., *p* = 0.007937, *n* = 5 in each group, the Mann–Whitney U test). Thus, this series of experiments clearly show that the PBM stimulates the FAβ evacuation from the hippocampus more effectively during sleep vs. wakefulness.

### Effects of 7 days-course of PBM during sleep and wakefulness on restoration of clearance of FAβ from the brain via the meninges after the MLV damage

**Fig. 9:**
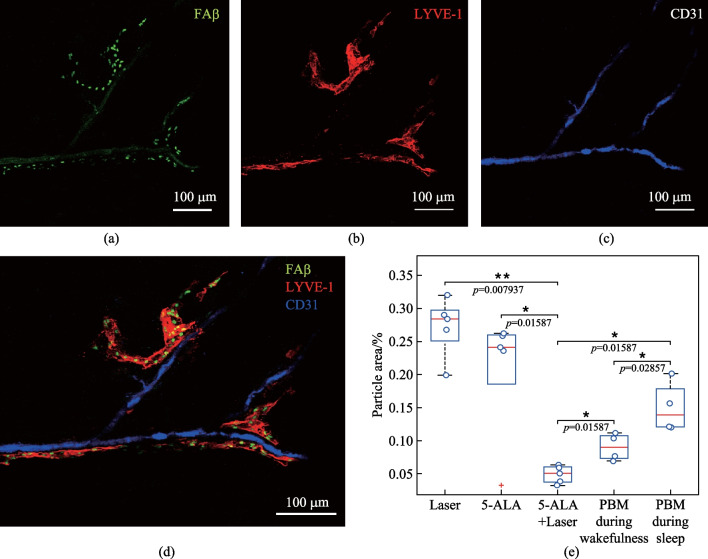
PBM effects during sleep and wakefulness on recovery of lymphatic removal of Aβ from the hippocampus after photo-ablation of MLVs: **a**–**d** removal of FAβ (green) via MLVs (red) labeled with LYVE-1, the blood vessels (blue) labeled with CD31; **e** quantitative analysis of FAβ presence in the meninges in the tested groups, (*n* = 5 in the groups “Laser,” “5-ALA,” “5-ALA + Laser”; *n* = 6 in the groups “PBM during wakefulness” and “PBM during sleep”), the Mann–Whitney U test

**Fig. 10:**
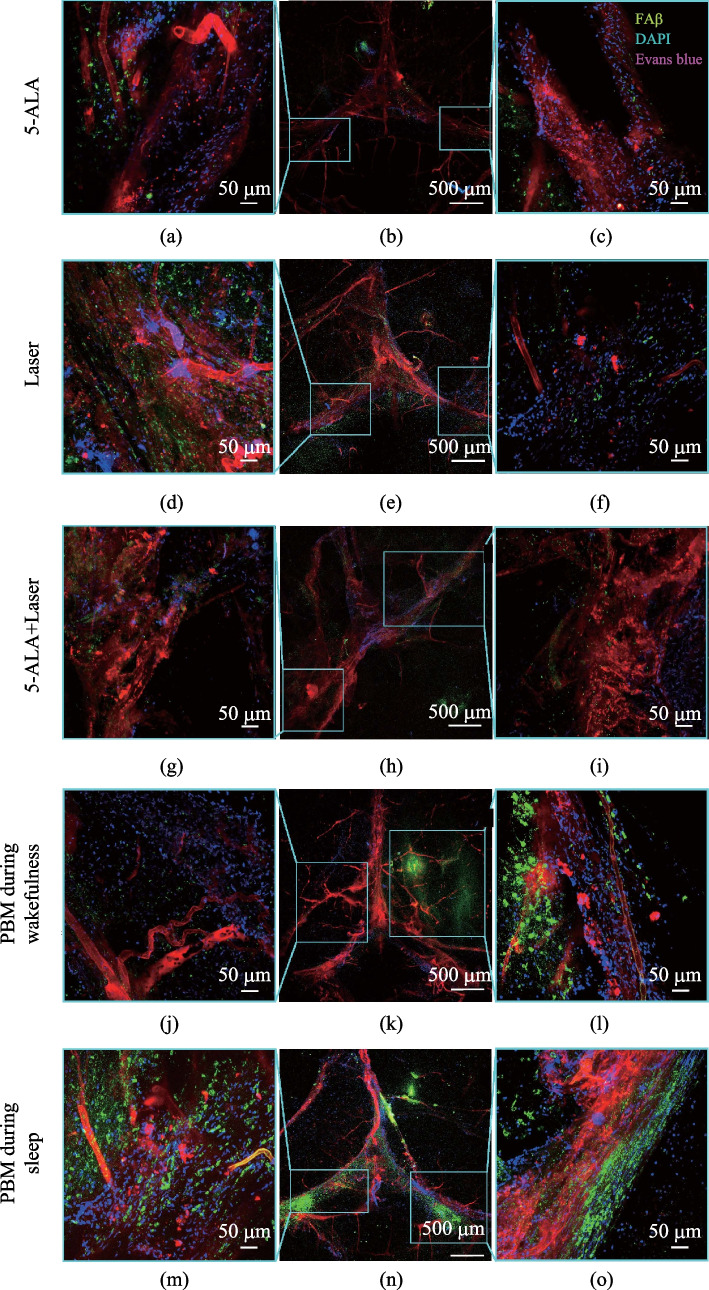
Confocal analysis of the PBM effects during sleep and wakefulness on recovery of removal of Aβ from the hippocampus via MLVs after their photo-ablation: **a**–**c** in the 5-ALA group; **d**–**f** in the Laser group; **g**–**i** in the 5-ALA + Laser group (photo-ablation of MLVs); **j**–**l** in the PBM during wakefulness group; **m**–**o** in the PBM during sleep group; green—FAβ; blue—DAPI; red—the blood vessels filled with Evans Blue

In the final step, we analyzed the effects of 7 days-course of PBM during sleep and wakefulness on removal of FAβ from the hippocampus via the meninges as an important route of clearance of Aβ from the brain [34, 38]. Indeed, Fig. 9a–d shows FAβ presence in the lumen of MLVs 3 days after its injection into the hippocampus suggesting that MLVs are pathway to remove FAβ from the brain. Figures 9e and 10 demonstrate the quantitative and qualitative analysis of the PBM effects during sleep and wakefulness on recovery of lymphatic removal of FAβ from the brain. The results clearly show that photo-ablation dramatically suppressed removal of FAβ from hippocampus that was restored after the 7 days-course of PBM more effectively when PBM was applied during sleep than during wakefulness. Indeed, the intensity of fluorescent signal from FAβ in the meninges was 4.60-fold and 4.20-fold lesser in the group “5-ALA + laser” vs. the group “Laser” and the group “5-ALA,” respectively (0.05 ± 0.005 a.u. vs. 0.23 ± 0.035 a.u., *p* = 0.007937 between the groups “5-ALA_Laser” and “Laser”; 0.05 ± 0.005 a.u. vs. 0.21 ± 0.006 a.u., *p* = 0.01587, between the groups “5-ALA_Laser” and “5-ALA,” *n* = 5 in each group, the Mann–Whitney U test). These data suggest the significant suppression of removal of Aβ from the brain via the meninges after the MLV injury.

At the same time, the intensity of fluorescent signal from FAβ was 3.00-fold and 1.80-fold higher in the groups “PBM during sleep” and “PBM during wakefulness” vs. the group “5-ALA + Laser, respectively (0.05 ± 0.005 a.u. vs. 0.15 ± 0.019 a.u., *p* = 0.01587 between the groups “5-ALA + Laser” and “PBM during sleep”; 0.05 ± 0.005 a.u. vs. 0.09 ± 0.010 a.u., *p* = 0.01587 between the groups “5-ALA + Laser” and “PBM during wakefulness”; *n* = 5 in the groups “5-ALA + Laser” and “Laser,” *n* = 4 in the groups “PBM during sleep” and “PBM during wakefulness,” the Mann–Whitney U test). Thus, PBM during sleep improved clearance of Aβ via the meninges better than in the waking state. Indeed, the intensity of fluorescent signal from FAβ in the meninges was 1.66-fold higher in the group “PBM during sleep” than in the group “PBM during wakefulness” (0.15 ± 0.019 a.u. vs. 0.09 ± 0.010 a.u., *n* = 4 in each group, *p* = 0.02857, the Mann–Whitney U test).

## Discussion

In this study, we answered to the question of why PBM during sleep is more effective in the AD therapy than during wakefulness. Since the brain lymphatics plays an important role in removal of Aβ from the brain and this system is activated during sleep [21, 22, 39–44], we tested our hypothesis that PBM can stimulate clearance of Aβ from the brain via the lymphatics stronger during sleep vs. wakefulness.

We found the presence of Aβ in MLVs after its injection into the hippocampus. These results confirm other data suggesting that MLVs are the tunnels for lymphatic transport of Aβ [22, 38]. In 1988, the Aβ protein was initially isolated from the homogenates of meninges from patients with AD [34]. Later, Da Mesquita et al. reported an important role of MLVs in removal of the Aβ plaques from the mouse brain with AD [38]. Thus, our and other data clearly demonstrate the lymphatic pathway of transport of Aβ from the brain.

In the experiments with photo-ablation of MLVs, we revealed that the injury of lymphatic vessels significantly suppresses distribution of FAβ in the brain tissues and the meninges as well as its evacuation from the hippocampus in mice. In our previous studies, we also show that photo-ablation of MLVs is associated with the significant decrease in lymphatic clearance of liposomes from the mouse brain [56]. Our current and early data clearly demonstrate that lymphatic route is necessary for clearance of different substances, including Aβ, from the brain.

We also demonstrate the effectiveness of new methods for photo-ablation of MLVs using PDT (5-ALA and laser 635 nm). Da Mesquita et al. proposed for photo-injury of MLVs to use photo-activation of Visudyne by a laser 689 nm [38]. In our previous work, we confirmed the effectiveness of this method for photo-damages of MLVs [56]. However, PDT-mediated injury of vessels is not specific and based on destructive effects of singlet oxygen and reactive oxygen species produced by photosensitizes after their photoexcitation [57]. Therefore, in this work, we used PDT with 5-ALA because this photosensitizer is used in clinical practice for PDT of glioblastoma [58, 59]. Recently, we reported that PDT with 5-ALA causes dose- and age-related injuries of cerebral blood vessels leading to an increase in the permeability of blood–brain barrier [52, 60–62]. Here, we demonstrate that PDT causes also damages of MLVs that is an important in our better understanding of the vascular effects of PDT.

The deep sleep is associated with activation of brain lymphatic drainage facilitating the elimination of metabolites, wastes and toxins from the human and animal brains [21, 22, 39–42]. In our results, we discovered that PBM promotes restoration of lymphatic functions after injury of MLVs that is more effective if PBM is used during deep sleep vs. wakefulness. Indeed, the evacuation of Aβ from the hippocampus and its subsequent distribution in the meninges after photo-ablation of MLVs were higher in mice that received PBM during deep sleep than mice treated by PBM during wakefulness. These data clearly demonstrate that PBM-mediated restoration of brain lymphatic function contributing to removal of Aβ from the brain is more effective during deep sleep that in the waking state. In our previous results, we showed that the memory recovery in mice with AD was more effective after the course of PBM during sleep than during wakefulness [22]. Recently, we reported that PBM increases the permeability of the lymphatic endothelium via an increase in production of nitric oxide in the endothelial cells [25, 27, 63]. We also showed PBM-related modulation of the tone of the lymphatic vessels as well as the phases of their contractility and relaxation that promotes better lymphatic removal of red blood cells from the brain in mice with intraventricular hemorrhages [63]. Thus, PBM-mediated augmentation of brain lymphatic functions can be a crucial mechanism responsible for the therapeutic effects of PBM for AD via an increase in removal of Aβ from the brain via MLVs.

## Conclusion

In this study on mice, we clearly demonstrate that PBM can improve the brain lymphatic functions after the MLVs injury. We also discover that PBM during sleep provides better restoration of removal of Aβ from the brain than PBM at the waking state. Our results shed light on the mechanism of PBM and show the stimulating effects of PBM on the brain lymphatic drainage that promotes transport of Aβ via the lymphatic pathway. The better effects of PBM on the brain lymphatics in sleeping brain open a new niche in the study of restorative functions of sleep and is an important informative platform for the development of innovative technologies of smart sleep for therapy of AD. Since pharmacological therapy of AD have failed to show effectiveness and safety, PBM as a non-invasive and safe approach has the high prospects for implementation in clinical practice for the treatment of brain diseases associated with lymphatic disorders, such as AD, Parkinson’s diseases, glioma, traumatic brain injury, intracranial hemorrhages [64–68].

## Data Availability

The data that support the findings of this study are available on request from the corresponding author.

## Footnotes

1 1 M = 1 mol/L.
